# Sequential Induction of Drug Resistance and Characterization of an Initial *Candida albicans* Drug-Sensitive Isolate

**DOI:** 10.3390/jof10050347

**Published:** 2024-05-13

**Authors:** Setrida El Hachem, Nour Fattouh, Christy Chedraoui, Marc Finianos, Ibrahim Bitar, Roy A. Khalaf

**Affiliations:** 1 Department of Natural Sciences, Lebanese American University, Byblos P.O. Box 36, Lebanon; setrida.elhachem@lau.edu (S.E.H.); nfattouh@sgub.edu.lb (N.F.); christy.chedraoui@lau.edu (C.C.); 2 Department of Biology, Saint George University of Beirut, Beirut 1100-2807, Lebanon; 3 Department of Microbiology, Faculty of Medicine, University Hospital in Pilsen, Charles University, 32300 Pilsen, Czech Republic; finianom@lfp.cuni.cz (M.F.); ibrahimbitar5@gmail.com (I.B.); 4 Biomedical Center, Faculty of Medicine, Charles University, 32300 Pilsen, Czech Republic

**Keywords:** drug resistance, ergosterol, chitin, biofilm, whole-genome sequencing

## Abstract

Background: The pathogenic fungus *Candida albicans* is a leading agent of death in immunocompromised individuals with a growing trend of antifungal resistance. Methods: The purpose is to induce resistance to drugs in a sensitive *C. albicans* strain followed by whole-genome sequencing to determine mechanisms of resistance. Strains will be assayed for pathogenicity attributes such as ergosterol and chitin content, growth rate, virulence, and biofilm formation. Results: We observed sequential increases in ergosterol and chitin content in fluconazole-resistant isolates by 78% and 44%. Surface thickening prevents the entry of the drug, resulting in resistance. Resistance imposed a fitness trade-off that led to reduced growth rates, biofilm formation, and virulence in our isolates. Sequencing revealed mutations in genes involved in resistance and pathogenicity such as *ERG11*, *CHS3*, *GSC2*, *CDR2*, *CRZ2*, and *MSH2*. We observed an increase in the number of mutations in key genes with a sequential increase in drug-selective pressures as the organism increased its odds of adapting to inhospitable environments. In *ALS4*, we observed two mutations in the susceptible strain and five mutations in the resistant strain. Conclusion: This is the first study to induce resistance followed by genotypic and phenotypic analysis of isolates to determine mechanisms of drug resistance.

## 1. Introduction

*Candida albicans* is a ubiquitous polymorphic opportunistic eukaryotic fungal pathogen that can cause a range of infections, from mild cases of oral thrush to severe systemic candidiasis [1]. One of the most common causes of candidemia—fungal bloodstream infection—is *C. albicans*, the fourth most common pathogen found in hospitals, and the leading fungal pathogen [2]. *C. albicans* has the ability to switch between different morphologies, from budding yeast to filamentous hyphae and pseudohyphae [3]. This switching ability is important for *C. albicans* to adapt to different environmental conditions in the host and thus become more virulent [4].

The cell wall of *C. albicans* is a dynamic organelle that is crucial for the microorganism’s survival as it confers shape, rigidity, and protection from the outer environment [5]. As the first structure that contacts the host, the cell wall is an important antigenic determinant. The main rationale of treatment in both superficial and disseminated candidiasis is via the administration of antifungal drugs. *C. albicans* and its host are eukaryotic, sharing high levels of gene orthology, rendering the development of new antifungals a difficult task [6]. In this study, we will be focusing on two classes of antifungal drugs, azoles and echinocandins. Azoles, such as fluconazole and voriconazole, hinder the production of ergosterol, which is a vital element in the fungal cell membrane [7]. The newest class of antifungals, echinocandins, like caspofungin, micafungin, and anidulafungin, inhibit the enzyme beta-(1,3)-D-glucan synthase. This enzyme is crucial for the synthesis of a vital component of the cell wall in numerous fungi [8].

Recently, the increased development of fluconazole resistance in *C. albicans* is associated with extensive clinical use. Different mechanisms are involved in resistance to fluconazole, such as mutations in *ERG11,* which results in an altered Erg11p structure with a decreased affinity to azoles, preventing them from binding to Erg11p. As such, ergosterol synthesis is no longer inhibited [9]. Another mechanism of resistance to fluconazole is through the overexpression of multi-drug resistance transporters (MDRs) that have a role in pumping the drugs out of the cell [10]. In addition, *ERG11* overexpression, resulting in high levels of plasma membrane ergosterol, is also a mechanism of resistance to fluconazole used by *C. albicans* to evade the immune system. An increase in Erg11p will allow the cell to tolerate higher levels of the drug and thus lower drug efficacy [11]. Moreover, *UPC2* gain-of-function mutation leads to reduced susceptibility of *C. albicans* to azoles. Upc2 is a transcription factor that is involved in the ergosterol biosynthesis pathway.

Resistance to echinocandins is mostly due to mutations in the *FKS1*, *FKS2,* and *FKS3* genes. These ortholog genes code for the catalytic subunit of 1,3-β-glucan synthase. Mutations in the binding domains that alter protein structure prevent the drug from binding, leading to echinocandin resistance, or inactivate the enzyme, resulting in a compensatory effect through chitin production, increasing the thickening of the cell wall and resulting in resistance [12,13].

As the evolution of drug resistance is a complex mechanism involving genotypic and phenotypic changes, our study aims to induce drug resistance to fluconazole and caspofungin in a sensitive *Candida albicans* SC5314 reference strain by growing the strain in increasing drug concentrations to better elucidate such mechanisms. Pathogenicity-related phenotypic attributes such as virulence, biofilm formation, ergosterol levels, and chitin levels will be determined in strains with various degrees of resistance. Genotypic changes will be addressed through whole-genome sequencing of sequentially resistant isolates to determine key mutations in genes that are involved in such resistance and pathogenicity attributes.

## 2. Materials and Methods

### 2.1. Candida albicans Isolates

Fluconazole or caspofungin was used for resistance induction in a susceptible reference strain, *C. albicans* SC5314 (ATCC^®^MYA-2876). A single, randomly selected colony of the susceptible reference strain was inoculated overnight in potato dextrose broth (Conda Laboratories, Madrid, Spain) at 30 °C in a shaking incubator at 100 rpm. After incubation, the cell count was adjusted to 10^6^ cells/mL in 1 mL of Roswell Park Memorial Institute (RPMI-1640) medium (Sigma-Aldrich, St. Louis, MO, USA) containing 0.19 µg of either fluconazole (Thermo Scientific, Waltham, MA, USA) or caspofungin (Sigma-Aldrich, St. Louis, MO, USA). The cells were incubated for two weeks in the conditions described above. The cell density was monitored and continuously diluted to 10^6^ cells/mL in fresh RPMI medium containing the aforementioned drug concentration. The drug concentration was then serially increased. Over the course of 3 months, the drug concentration was gradually increased up to 16 µg/mL for fluconazole (Table 1) and 2 µg/mL for caspofungin (Table 2). Isolates were labelled as susceptible, intermediate, resistant, and two-fold resistant according to the Clinical and Laboratory Standard Institute and stored in 15% glycerol at −80 °C.

### 2.2. DNA Extraction

DNA was extracted from freshly grown colonies using the ZR Fungal/Bacterial DNA MiniPrep^TM^ (Zymo Research, Irvine, CA, USA) kit according to the manufacturer’s instructions, with one modification. The elution buffer was switched out for one that did not contain EDTA.

### 2.3. DNA Sequencing and Sequence Analysis

DNA was sent for whole-genome Illumina sequencing at 90× coverage and sequence assembly to MicrobesNG, University of Birmingham, UK. Using the most suitable reference sequence, variant calling was performed by MicrobesNG to predict variants. The data were visualized and analyzed using the Integrative Genomics Viewer software v2.16.2. Sequences were deposited in the NCBI database under BioProject ID PRJNA1088846.

#### 2.3.1. Single-Nucleotide Polymorphism Detection

SNPs of the SC5314 *C*. *albicans* genomes were compared to the SNPs of the isolates by using the snippy multicommand (snippy-base application v4.5.0) [14] that generates a core genome multiple alignment against a common reference. The *C. albicans* SC5314 was used as a reference. The pipeline detects the variants and generates a single file for each isolate, listing the different variations.

#### 2.3.2. Annotation

Gene prediction was achieved using the BRAKER2 v2.1.6 pipeline in fungus mode, which combines GeneMarK-ES v4.65 and AUGUSTUS 3.4.0 for fungal gene prediction and identifying gene locations with the corresponding CDS and messenger RNA (mRNA) qualifiers [15,16,17]. Interproscan 5.50-84.0 [18] was used on the COG database to create the xml file to further incorporate in the functional annotation pipeline created by Funannotate 1.8.7 [19,20]. The pipeline starts by running HMMscan (HMMer v3.3) (hmmer.org) with default parameters on the PFAM database, then using emapper 2.1.2 based on eggnog orthology data [21]. Sequence searches were performed using Diamond Blastp v.0.4.7 [22] on UniProt DB version 2021_02 and MEROPS v12.0; the resulting annotations were combined using Gene2Product v1.69, and later, Signalp 5.0 was used to predict secreted proteins [23]. Assemblies and annotations were assessed using BUSCO V5.2.2 [24].

### 2.4. Quantification of Ergosterol Content in the Plasma Membrane

One colony of each isolate was inoculated into 50 mL of potato dextrose both and incubated for 17 h in a shaking incubator at 100 rpm and 35 °C. The sterol quantification method followed. Ergosterol was extracted from the grown cells and quantified using the methods and equations found in [25]. In summary, the *C. albicans* culture was centrifuged at 2700 rpm for 5 min and the pellet was weighed, dissolved in 3 mL of a 25% alcoholic potassium hydroxide solution, vortexed for 1 min, and transferred to a glass screw-cap tube. The suspended pellet was incubated at 85 °C for 1 h in a water bath, cooled down at room temperature, mixed with 3 mL of *n*-heptane and 1 mL of distilled water, vortexed for 3 min, and incubated for 1 h at room temperature. Upon phase separation, the *n*-heptane layer was transferred to another glass screw-cap tube and incubated for 24 h at −20 °C. The *n*-heptane layer was diluted 5-fold in 100% ethanol. The optical density was measured using a Genesys 10S UV-Vis spectrophotometer (Thermo Scientific, Waltham, MA, USA) at 230 and 281.5 nm. Each isolate’s ergosterol content was quantified in biological triplicates and the average percent of ergosterol (erg.) was computed. Then, the percentage change in ergosterol (erg.) was calculated for each isolate compared to the *C. albicans* SC5314 isolate according to the formula below:

%change in erg. content = [(% erg. in isolate − % erg. in SC5314)/% erg. in SC5314] × 100

### 2.5. Virulence Assay

For the virulence assay, 10^7^ cells in 200 µL of 1× phosphate-buffered saline of each isolate, including the reference SC5314 strain, were injected into the tail vein of six 4-week-old, non-pathogen-free BALB/c female mice to induce a systemic infection. A control group of 6 mice was injected with only 1× phosphate-buffered saline solution [1,13]. The mice were monitored for 30 days and the number of moribund mice was counted daily. Moribund mice were euthanized. Protocols followed the ethical standards of the Lebanese American University’s Institutional Animal Care and Use Committee. This study was conducted under approval number IRB #: LAU.ACUC.SAS.RK3.28/January/2022.

### 2.6. Biofilm Formation Assay

One colony of each isolate was inoculated into 5 mL of potato dextrose broth at 30 °C in a shaking incubator at 110 rpm overnight. The optical density was measured using a Genesys 10S UV-Vis spectrophotometer at 660 nm; 200 µL of each sample was placed into a 96-well plate, with the number of cells adjusted to 2 × 10^6^ cells/mL. The plate was put in an 80 rpm shaking incubator at 37 °C for 3 h. The wells were refreshed with 200 µL of new potato dextrose broth and placed in a non-shaking incubator at 37 °C for 48 h. Biofilm formation was evaluated based on the crystal violet assay protocol described in [26] with some modifications. The potato dextrose broth was discarded from the wells. Then, 200 µL of 99% methanol was added and left at room temperature for one hour. The methanol was removed and the plate was left to air dry for 45 min. Next, 200 µL of 0.01% crystal violet was added to each well, left for 30 min at room temperature, and then washed 5 times with sterile water. To quantify the biofilm, 200 µL of 33% acetic acid was added to each well and the optical density in each well was measured by the Multiskan TM FC Microplate Photometer (Thermo Fisher Scientific, Rockford, IL, USA)and the SkanIt TM Software v2.0 for microplate readers at 595 nm. The experiment was performed in biological triplicates.

### 2.7. Quantification of Cell Wall Chitin

One colony of each isolate was grown in 10 mL of potato dextrose broth in a shaking incubator at 100 rpm and 30 °C. Chitin was extracted and quantified from the cell wall using the methods in the protocol described in [1] with some modifications. *C. albicans* cultures were centrifuged for 5 min at 4000 rpm. The pellets were resuspended in 500 µL of 5 mM Tris (pH 7.8) and transferred to 2 mL Eppendorf tubes with a 1:1 refrigerated glass bead-to-pellet ratio. The samples were vortexed for 1 h and then poured into new 1.5 mL Eppendorf tubes. The beads were washed with 50 µL of refrigerated 1 M NaCl and then mixed with the respective samples to prevent sample loss. The Eppendorf tubes were centrifuged at 3000 rpm for 5 min and the pellets were weighed. Cell wall proteins were eliminated by resuspending the pellets in a protein extraction buffer (150 mM NaCl, 100 mM Na-EDTA, 50 mM Tris buffer, 2% SDS, 8 µL/1 mL β-mercaptoethanol, pH 7.8) at a ratio of 500 µL per 100 mg of pellet weight. Tubes were boiled at 99 °C for 5 min, cooled down for 5 min, and centrifuged for 5 min at 3000 rpm. This step was performed two times. Pellets were washed 3 times by resuspension in sterile water and then centrifuged for 5 min at 3000 rpm. Pellets were suspended in 1 mL of 6 N HCl and boiled at 99 °C for 10 min, centrifuged for 5 min at 3000 rpm, and then suspended in 1 mL of sterile water. The quantification of chitin was performed by following the protocol described in [27]. The experiment was performed in biological triplicates.

### 2.8. Growth Kinetics

One colony of each *Candida albicans* isolate was inoculated into 2 mL of potato dextrose broth in a shaking incubator at 30 °C and 100 rpm. Then, 8 mL of fresh potato dextrose broth was added to each of the overnight cultures and incubated for 2 h in the same conditions. The optical density was measured using a Genesys 10S UV-Vis spectrophotometer at 660 nm to prepare 10 mL potato dextrose cultures with 10^4^ refreshed *C. albicans* cells/mL. The cultures were incubated in a 30 °C shaking incubator at 100 rpm for 10 h. The optical density was measured every 2 h. The experiment was performed in biological triplicates for each isolate. The values were averaged to generate a growth curve over the course of 10 h.

### 2.9. Statistical Analysis

Statistical analysis was performed using the GraphPad Prism version 7.00 software. For the ergosterol content quantification, biofilm formation assay, and chitin quantification assay, a Friedman test and Dunn’s multiple comparison test were performed to compare the data of all isolates. An ordinary two-way ANOVA and Dunnett’s multiple comparison tests were performed for the growth kinetics. The data were compared to those of the SC5314 reference strain. Data were only considered significant if *p*-values were below 0.05.

## 3. Results

### 3.1. Ergosterol Quantification Assay

**Figure 1:**
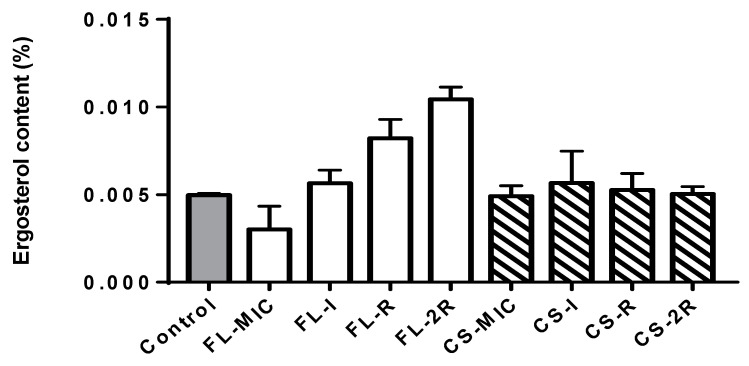
Ergosterol content. The grey bar represents the control strain that was not grown in any concentration of drugs. White bars represent the isolates that were grown in increasing concentrations of fluconazole. The striped bars represent the isolates grown in increasing concentrations of caspofungin. Statistical significance was calculated using Friedman and Dunn’s multiple comparisons tests where the *p*-value of fluconazole isolates is less than 0.0001, and that for caspofungin is *p* = 0.9137.

The ergosterol content increased throughout the induction of resistance to fluconazole. The resistant isolate showed a notable increase in ergosterol content with a 65% increase compared to the control strain. A further increase was seen in the isolate exhibiting two-fold resistance (2R) to fluconazole with a 78% increase compared to the control and a *p*-value of 0.0017. However, for the isolates grown in caspofungin, there was no significant difference between the percentages of ergosterol between the isolates as caspofungin does not target ergosterol synthesis; thus, the ergosterol content was similar to the control (Figure 1).

### 3.2. Chitin Quantification Assay

**Figure 2:**
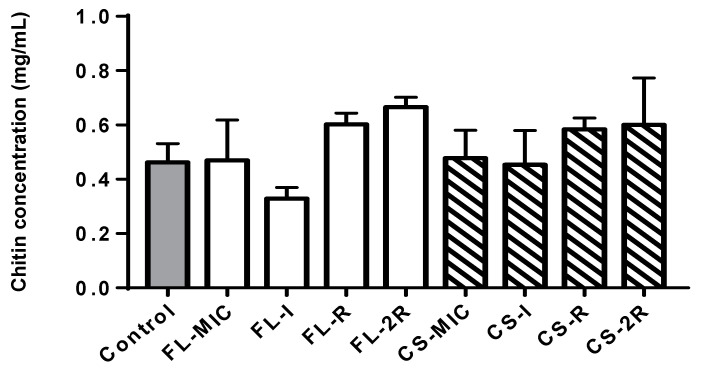
Chitin content. The grey bar represents the control strain that was not grown in any concentration of drugs. White bars represent the isolates that were grown in increasing concentrations of fluconazole. The striped bars represent the isolates grown in increasing concentrations of caspofungin. Values of chitin concentration in 100 mg of weight pellet were used. Statistical significance was calculated using Friedman and Dunn’s multiple comparisons tests where the *p*-value of fluconazole isolates is 0.004 and that for caspofungin is *p* = 0.4056.

The chitin content increased throughout the induction of resistance to caspofungin. The susceptible isolate grown in a concentration of drugs equal to the MIC showed a 3% increase in chitin compared to the control. However, the isolate that exhibited resistance to caspofungin showed an increase in chitin content, with a 26% increase compared to the control strain. Moreover, a further increase was seen in the isolate exhibiting two-fold resistance (2R) to caspofungin with a 30% increase compared to the control. Similarly, for the fluconazole-treated isolates, there was also a significant increase in chitin content, with the R isolate having a 30% increase compared to the control and the 2R isolate having a 44% increase compared to the control with a *p*-value of 0.004 (Figure 2).

### 3.3. Biofilm Quantification Assay

**Figure 3:**
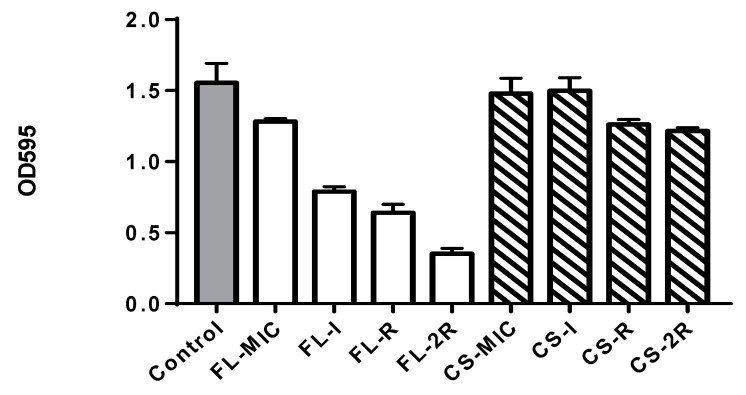
Biofilm quantification. The grey bar represents the control strain that was not grown in any concentration of drugs, white bars represent the isolates that were grown in increasing concentrations of fluconazole, and the striped bars represent the isolates grown in increasing concentrations of caspofungin. Statistical significance was calculated using Friedman and Dunn’s multiple comparisons tests; for fluconazole isolates, *p* < 0.0001, and for caspofungin, *p* = 0.0151.

A significant decrease in biofilm formation was noticed for fluconazole-treated isolates (Figure 3) with a *p*-value less than 0.0001. The control isolate had an OD595 of 1.554, FL-MIC had an OD595 of 1.282, FL-I was 0.789, and FL-R was 0.640, and finally, the OD595 of FL-2R was measured as 0.352. The caspofungin-treated isolates showed a significant decrease in biofilm formation with a *p*-value of 0.01, starting with the control OD595 equal to 1.554 and ending with the resistant isolate CS-2R exhibiting an OD equal to 1.216 (a decrease of 21.76%). Such a decrease has been reported previously.

### 3.4. Growth Rate Assay

**Figure 4:**
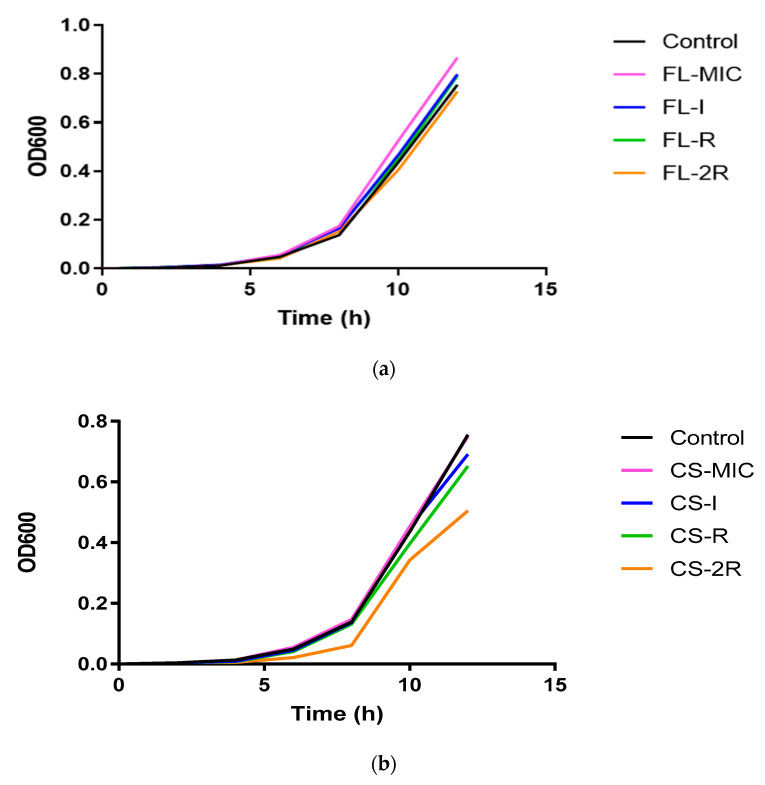
(**a**) Growth rate for fluconazole isolates. This chart represents the growth rates of fluconazole-treated isolates monitored every 2 h over the course of 12 h. (**b**) Growth rate for caspofungin isolates. This chart represents the growth rates of caspofungin-treated isolates monitored every 2 h over the course of 12 h.

In Figure 4a, it can be seen that there was a slight decrease in the growth rates of fluconazole-treated isolates where the susceptible isolate (pink line) had an OD at 600 nm of 0.867 after 12 h, whereas for isolate 2R, the OD was 0.728 (orange line).

Similarly, in Figure 4b, there was a larger decrease in the growth rate between the susceptible isolate of caspofungin and the resistant isolate, where the OD600 of CS-MIC was 0.750 (pink line) while the OD600 of 2R was 0.505 (orange line).

This could be due to an increase in cell wall chitin thickness observed in the resistant isolates, affecting cell division. An altered, thick cell surface composition can disrupt normal cellular functions, including nutrient uptake and cell division, resulting in decreased growth rates [28].

### 3.5. Virulence Assay

**Figure 5:**
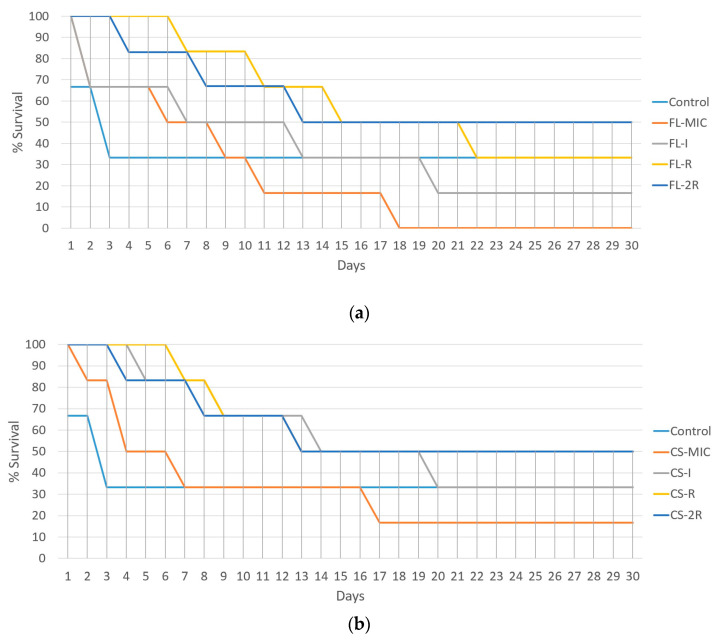
(**a**) Virulence assay for fluconazole-resistant isolates. The percent survival of mice post-injection is represented as a function of days. Control is the group of mice that were injected with the *C. albicans* reference strain that was used for resistance induction. Each group consists of 6 injected mice. (**b**) Virulence assay for caspofungin-resistant isolates. The percent survival of mice post-injection is represented as a function of days. Control is the group of mice that were injected with the *C. albicans* reference strain that was used for resistance induction. Each group consists of 6 injected mice.

Over the course of 30 days, the control strain experienced 4 deaths, the FL-MIC strain experienced complete mortality (all six mice), FL-I had five deaths, FL-R had four deaths, and FL-2R had three deaths. In the case of caspofungin-treated isolates, CS-MIC resulted in fivedeaths, CS-I had four, and both CS-R and CS-2R resulted in threedeaths (Figure 5).

### 3.6. WGS Analysis

#### 3.6.1. Fluconazole Isolates

Based on the variant calling data obtained, a table (Table 3) was generated for the fluconazole-treated isolates. Sixty genes involved in resistance, biofilm formation, immune evasion, phenotypic switching, and stress response were analyzed and compared, and twenty-one genes were found to have a sequential increase in mutations. These genes are reported in the table below and grouped in order of function.

#### 3.6.2. Caspofungin Isolates

Based on the variant calling files, a table (Table 4) was generated for the caspofungin-treated isolates. Fifty genes involved in resistance, biofilm formation, immune evasion, phenotypic switching, and stress response were closely analyzed and compared, and eighteen genes were found to contain a sequential mutation increase. These genes are reported in the table below and grouped in order of function. A asterisk sign next to the gene indicates that it was previously reported.

## 4. Discussion

The acquisition of resistance to fluconazole and caspofungin in *C. albicans* is a phenomenon that involves complex mechanisms, some of which are not well understood. Resistance often leads to significant shifts in the fungal cell structure and physiology along with mutations in the genome. Therefore, the objective of this study was to induce resistance to a susceptible strain of *C. albicans* in vitro in a lab setting and analyze the different phenotypes and genotypes obtained while acquiring resistance to two drugs, fluconazole and caspofungin. In addition, our study also aimed to address the sequential evolution of resistance, whether intermediary-resistant isolates exhibit intermediary phenotypes, and whether the accumulation of mutations goes hand in hand with an increased acquisition of resistance.

In this study, we scanned genes involved in drug resistance, stress response, phenotypic switch, biofilm formation, and immune evasion for mutations to confirm the phenotypes seen in the experimental part of our study. *ERG11*, *CDR2*, *MSH2*, *CHS3*, *ERG4*, *SOD5*, *SOD6*, *HSP60*, *MNL1*, *SIR2*, *SAP1*, *CPH1*, *EFG1*, *MFG1*, *RFG1*, *FGR15*, *HWP1*, *ALS4*, *NAG3*, *UME6*, *SFL1*, *HSP60*, *GSC2*, *CRZ2*, and *ATG26* were the genes that we examined in our WGS analysis. Mutational analysis and SNP identification were carried out by MicrobesNG. In addition, an independent evaluation of the variant calling process was carried out to ensure the accuracy and reliability of the identified variant through Snippy multicommand tools. The details of the variant calling procedure are described in the methods section.

A significant increase in ergosterol content was reported in this study. This component is vital for the fungal cell membrane, and differences in its concentration can directly affect the susceptibility of *C. albicans* to antifungal agents, specifically to azoles such as fluconazole. Interestingly, we did not observe any difference in ergosterol concentrations for the caspofungin isolates. This is documented in the literature, as only a relationship between the ergosterol pathway and fluconazole resistance is documented [29,30]. As such, in our study, a significant 65% increase in ergosterol content was observed in the fluconazole-resistant isolates compared to the control, indicating that *C. albicans* developed a compensatory mechanism by upregulating ergosterol biosynthesis, resulting in a thickening of the cell surface, which makes it harder for the drug to permeate the cell, hence maintaining cell membrane integrity. This phenotypic mechanism of acquisition of resistance was mirrored in the genotypic analysis through whole-genome sequencing data. Missense mutations were observed in the *ERG11* gene of the resistant phenotypes, providing valuable insights into the mechanism behind the observed phenotypes. One of the amino acid substitutions in the *ERG11* gene was previously reported to be involved in the development of resistance to fluconazole [11,31]. By contrast, the L264S mutation was not previously documented. Further analysis should be carried out in order to understand its exact role in resistance. However, point mutations that change the tertiary structure of the enzyme and prevent drug binding are a known mechanism of resistance. Moreover, point mutations accumulating sequentially throughout the acquisition of resistance to fluconazole were seen in the *CDR1* gene, and none of these mutations were previously reported in the literature. Mutations in *CDR1* can upregulate efflux pumps that pump the drug out of the cell, resulting in resistance.

For isolates that exhibited resistance to caspofungin, the ergosterol content showed no difference amongst them. This observation aligns with the fact that the acquisition of resistance to caspofungin does not target the ergosterol synthesis pathway. Nevertheless, within the caspofungin-resistant isolates, chitin levels did increase, but not significantly, whereas a significant yet perhaps unexpected finding is that a 44% increase in chitin content was reported in the fluconazole-resistant isolates, as chitin increase is usually associated with echinocandin resistance, not azole resistance, suggesting a cross-talk between the two pathways. As mentioned previously, chitin is a crucial structural component of the fungal cell wall, and its modulation shows an adaptive response to the new environment with a high drug concentration. The increase in chitin content helps in cell wall reinforcement, cell integration, and counteracting the damaging effects of the drugs. This increase in chitin content was confirmed by the WGS data of fluconazole isolates, where an accumulation of mutations in *CHS3* were observed in the fluconazole-resistant strains, which is the main enzyme involved in cell wall chitin synthesis, and mutations in this gene have been previously associated with increased chitin deposition [32]

Moreover, this study shows a significant decrease in biofilm formation with increasing resistance to both fluconazole and caspofungin. Mutations in genes that lead to alterations in the cell wall and decreased adhesion will impact the ability of *C. albicans* to form biofilms and adhere to surfaces [33]. As observed, the chitin content in the fluconazole-resistant isolates increased significantly compared to the caspofungin-resistant isolates; this observation aligns with the high reduction in biofilm formation for the fluconazole-resistant isolates compared to the caspofungin-resistant isolates that exhibited a modest decrease in biofilm formation. Increased chitin alters the cell wall architecture and cell wall protein deposition, preventing proper biofilm formation. This was further confirmed by WGS data that showed mutations in *HWP1* and *ALS4* genes, affecting adhesion and biofilm formation in fluconazole-resistant isolates.

*C. albicans* responds to antifungal drug exposure by activating stress response pathways. The activation of these pathways diverts cellular resources towards stress adaptation and defence mechanisms, which induce cell cycle arrest and delay cell division, leading to a decreased growth rate [34]. Stress response-related genes such as *SOD5*, *SOD6*, *HSP60*, *MNL1*, *SIR2*, and *ATG26* were found to be mutated in both fluconazole- and caspofungin-treated isolates. Our virulence assay data showed a decrease in virulence with increased resistance. This has been reported in the literature, whereby acquisition or resistance comes at a cost of loss of fitness through decreased virulence. In fact, our FL-MIC and CS-MIC isolates showed the highest number of mortalities compared to the more resistant isolates. Thickening of the cell wall through increased chitin deposition and increased ergosterol synthesis might hinder proper adhesion, cell division, or filamentation [13]. Lack of adhesion is reflected as an attenuation of virulence, since adhesion is the first and necessary step in virulence This was also reflected in our growth assays, whereby more resistant isolates had an increased doubling time. Our WGS data support this, as genes involved in filamentation and virulence-associated factors such as *SAP1*, *CPH1*, *MFG1*, *FGR15*, *SFL1*, and *NAG3* exhibited multiple deletions and point mutations.

Another interesting aspect of our study is that a sequential increase in resistance was mirrored by a sequential accumulation of mutations over time. For example, only one mutation was observed in *ERG11* at fluconazole MIC concentrations as opposed to three in the 2R isolate. *CHS3* exhibited no mutations at low drug concentrations but three-point mutations at high drug concentrations. *ALS4,* involved in adhesion, went from two mutations to five with increased exposure. Microorganisms are well known for their genetic plasticity, allowing them to undergo rapid genetic changes in response to stressful environments such as drug exposure by expanding mutation rates to increase their odds of survival—a case-in-point example of natural selection.

Our work is the first of its kind to induce resistance in sensitive laboratory isolates followed by whole-genome sequencing and pathogenicity-related phenotypic assays. We were able to show that resistance and pathogenicity attributes, such as ergosterol and chitin content and biofilm-forming ability, were affected by exposure to the drug, implying that at the physiological level, the cell responded to drug exposure by modulating its cell surface. This change in physiology was reflected at the genomic level with mutations in key genes involved in resistance mechanisms. The idea of “accelerating evolution” is of clinical relevance since it gives scientists a glimpse of possible resistance mechanisms that might evolve in the future, and to start preparing all the necessary tools and antifungal drugs to combat novel resistance pathways.

Although this study helps clinicians and researchers improve their understanding of antifungal resistance development mechanisms to design appropriate prophylaxis and treatment strategies, four limitations need to be mentioned: (1) The induction of resistance was carried out over a short period of time in vitro compared to the natural selection that would take place in vivo and expose the fungus to the immune system. (2) In general, nosocomial *C. albicans* are exposed to multiple antifungal agents and not only one. (3) Transcriptomics was not carried out, so changes in the expression of genes involved in antifungal resistance, such as those coding for efflux pumps, were not quantified. (4) Mutations should be reintroduced by genetic modifications into a susceptible strain to determine whether the mutation is directly responsible for resistance.

## Figures and Tables

**Table 1:** Induction of resistance to fluconazole in *C. albicans.* Isolates were grown in the presence of increasing drug concentrations.

Isolate	Fluconazole Concentration
FL–MIC	0.190 µg/mL
FL–Int	4.095 µg/mL
FL–R	8.000 µg/mL
FL–2R	16.000 µg/mL

**Table 2:** Induction of resistance to caspofungin in *C. albicans.* Isolates were grown in the presence of increasing drug concentrations.

Isolate	Caspofungin Concentration
CS–MIC	0.190 µg/mL
CS–Int	0.595 µg/mL
CS–R	1.000 µg/mL
CS–2R	2.000 µg/mL

**Table 3:** WGS analysis and SNP identification. This table shows the different SNPs identified in the fluconazole-treated isolates for genes involved in drug resistance, stress response, phenotypic switch, biofilm formation, and immune evasion. Note the sequential accumulation of mutations in the same gene as the drug concentration increases.

		FL-MIC	FL-I	FL-R	FL-2R
Function	Genes	AA Change	AA Change	AA Change	AA Change
Drug Resistance	*ERG11*	L220L	L220L–L480L	L220L–L480L	L220L–T128K–L264S
	*CDR2*	I704M–R365Q	I704M–M617L	I704M–M617L	R365Q–I704M–E374K
	*MSH2*	D73D	T141T	V127	V127A
	*CHS3*	-	R762P	L321P–D388G–R762P	L321P–D388G–R762P
	*ERG4*	-	-	-	M157V
Stress response	*SOD5*	-	A210P	A210P	A210P–G214D
	*SOD6*	-	S225P	S225P	S225P
	*HSP60*	K67K	N281N	S267A	S267A
	*MNL1*	H349R	H349R–I175T	H349R–I175T	H349R–I175T
Phenotypic Switching/dimorphism	*SAP1*	L53	L53I	L53I	L53I
	*CPH1*	T510A	T510A	T510A	T510A
	*EFG1*	K251K	K251K	I384T	I384T
	*MFG1*	-	A68P	A68P–Q76_Q80del	A68P–Q76_Q80del
	*SIR2*	L213	L213	L213	L213S
	*RFG1*	-	-	Q118del	Q118del
	*FGR15*	V131A	V131A	V131A–S361L	V131A–S361L
Biofilm Formation and Immune Evasion	*HWP1*	-	-	T296A–S441P	T296A–S441P
	*ALS4*	I79V–V83I	I79V–V83I–V111A	I79V–V83I–V111A– S186P–D198N	I79V–V83I–V111A– S186P–D198N
	*NAG3*	-	R158*	Y278H	Y278H
	*UME6*	A146A	N257S–M267I	N257S–M267I	N257S–M267I –N396S
	*SFL1*	V691I	V691I	V691I–S373L	V691I–S373L

**Table 4:** WGS analysis and SNP identification. This table shows the different SNPs identified in the caspofungin-treated isolates for genes involved in drug resistance, stress response, phenotypic switch, biofilm formation, and immune evasion. Note the sequential accumulation of mutations as drug resistance increases.

		CS-MIC	CS-I	CS-R	CS-2R
Function	Genes	AA Change	AA Change	AA Change	AA Change
Drug Resistance	*CDR2*	I704M	I704M–M617L	I704M–M617L–E374K	I704M–M617L–E374K–R365Q
	*CRZ2*	-	-	S330A	S330A
	*GSC2*	A455A	A462P	A462P	A462P
Stress response	*SOD5*	-	A210P	A210P–G214D	A210P–G214D
	*SOD6*	-	S225P	S225P	S225P
	*HSP60*	S267A	S267A	S267A	S267A
	*MNL1*	H349R	H349R–I175T	H349R–I175T	R269 *
	*ATG26*	-	Y401C	E269K–Y401C	E269K–Y401C–T414I
Phenotypic Switching/dimorphism	*CPH1*	P451P	I79I	T510A	T510A
	*MFG1*	-	-	Q80del	Q80del
	*RFG1*	-	-	T39S	T39S
	*SIR2*	-	-	L213S	L213S
	*FGR15*	-	S361L	S361L	S361L
Biofilm Formation and Immune Evasion	*HWP1*	-	S441P	S441P–T296A	S441P–T296A
	*ALS4*	-	I79V–V83I–V111A	I79V–V83I–V111A–S186P	I79V–V83I–V111A–S186P–D198N
	*NAG3*	-	-	Y278H	R158 *
	*UME6*	N257S–M267I	N257S–M267I	N257S–M267I–N396S	N257S–M267I–N396S–N484del
	*SFL1*	V691I–S373L	V691I–S373L	V691I–S373L–T75A	T75A–Q301fs

## Data Availability

Sequences were deposited in the NCBI database under BioProject ID PRJNA1088846.
